# Salivary cortisol determination in patients from the Basque Country with recurrent aphthous stomatitis. A pilot study

**DOI:** 10.4317/medoral.18110

**Published:** 2013-02-05

**Authors:** Asier Eguia-del Valle, Rafael Martínez-Conde-Llamosas, José López-Vicente, Agurne Uribarri-Etxebarria, José M. Aguirre-Urizar

**Affiliations:** 1 Oral Medicine.Unit. Oral and Maxillofacial Pathology Unit. Faculty of Medicine and Dentistry. UFI 11/25.University of the Basque Country / EHU; 2 ……..; 3 ….

## Abstract

Objectives: Stress and anxiety are controversial factors involved in the complex pathogenesis of Recurrent Aphthous Stomatitis (RAS). The determination of salivary cortisol is a useful, simple and safe test to detect states of high stress or anxiety. The aim of this study is to check for changes in salivary cortisol levels in patients with RAS during periods of active disease. Study design: A measurement of cortisol employing Enzyme-Linked Immuno Sorbent Assay (ELISA) was carried out in samples of unstimulated saliva from 20 patients with active lesions of RAS and 10 healthy individuals used as controls. Results: Increased levels of salivary cortisol were detected in 3 cases, all of them within the group of patients with RAS. In none of the control group patients the level of salivary cortisol was increased. The mean level of salivary cortisol was 0.64 mg / dl (range 0.2 to 1.62) for patients with RAS and 0.57 mg / dl (range 0.25 to 1.09) for controls. Conclusion: Salivary cortisol levels are not statistically higher in patients with active lesions of RAS.

** Key words:**Recurrent aphthous stomatitis, cortisol, oral ulcers, canker sores, salivary cortisol.

## Introduction

Recurrent Aphthous Stomatitis (RAS) is one of the most common diseases of the oral mucosa (1). However, the pathogenesis of RAS is not yet completely understood despite its high frequency. RAS is also one of oral diseases on which more research work has been published in the medical literature (1). There is much controversy on its complex pathogenesis, with conflicting opinions between different groups of researchers (1,2). Many different factors such as genetic, immunological, microbiological, nutritional, hormonal, emotional, traumatic and others are involved in the etiology of RAS. Unfortunately, there is still no clear and definitive explanation about how all these factors are really implied in the pathogenesis of RAS (1,2).

Cortisol is the most abundant circulating steroid and the major glucocorticoid secreted by the adrenal cortex (3,4). Cortisol is involved in gluconeogenesis, calcium absorption and secretion of gastric acid and pepsin. Furthermore, it has an active role in the inflammatory response and in the regulation of blood pressure (3,4). The measurement of cortisol levels is also considered as a good indicator of adrenocortical function. Because of this, it is very useful in the diagnosis of Addison’s disease and Cushing, hypopituitarism and adrenal hyperplasia and carcinoma (3,4). Cortisol levels are abnormally increased in situations of pain, stress, acute infections, diabetes mellitus, heart failure, pregnancy and in patients under estrogen therapy (3,4).

Close correlations between serum and salivary levels of cortisol have been demonstrated in different studies (5,6). Therefore, salivary cortisol determination is currently considered a reliable indicator of adrenal function.

There are several processes that can modify the concentration of salivary cortisol. Some of them, such as depressive states can make it fall. However, others such as stress or anxiety states can increase it (7,8). Interestingly, patients with RAS often relate the onset of new lesions with what they recognize as “periods of increased stress or anxiety”.

Several authors (9-12) have studied the salivary levels of this glucocorticoid in different oral conditions such as lichen planus, xerostomia, periodontal disease and RAS, with no conclusive results obtained to date.

The aim of this study is to check if salivary cortisol levels are higher in patients with RAS during periods of active disease than in healthy individuals.

## Material and Methods

A prospective observational pilot study was performed on 20 patients with RAS selected by simple randomization (using tables of random numbers) from a group of patients previously diagnosed with RAS in the Service of Oral Medicine of the Dentistry Clinic of The University of the Basque Country / EHU. All the patients in this group attended regularly to control reviews and met the inclusion and exclusion criteria of RAS. Salivary samples were collected during the years 2007 and 2008, always during the presence of active lesions in ulcerative phase.

The study group was composed of 12 women (60%) and 8 men (40%) with a mean age of 35.9 years (mean deviation: ± 14.2, range: 21-67).

The control group consisted of 10 healthy individuals living in the Basque Country without RAS, 5 men and 5 women, with a mean age of 36.6 years (mean deviation: ± 11.9, range: 20-56). These patients were selected by simple randomization (with random number tables) from a group of people attending a dental check to the Dental Clinic of the University during the same period of time.

The criteria for inclusion in the group of patients with RAS were lived in the Basque Country and had a clear diagnosis of the disease, fulfilling the clinical characteristics that typically define the RAS (1,2).

Patients with severe systemic disease or immunodeficiency, endocrine or metabolic disease, and patients with other concomitant oral diseases were excluded from the study. Patients with inconclusive diagnosis of RAS were also excluded.

Unstimulated salivary samples were collected from all patients after carrying out a detailed medical history. Previously, the informed consent was obtained from all the patients. Salivary samples were collected asking the patients to deposit all their saliva into a test tube for five minutes (13). All of the samples were collected between 9 and 12 AM to minimize the effect of thecircadian cycle. In every case it was performed one hour and a half after awakening or food intake.

Salivary samples were frozen at -25 ° C temperature and stored, after cooling to room temperature for 10 minutes.

The measurement of salivary cortisol was obtained by Enzyme Linked Inmunosorbent Assay (ELISA) using the Immulite 2000 Cortisol® test (Diagnostic Product Corporation, Dipesa SA, Spain). The Automated Immulite 2000® analysis unit (Diagnostic Product Corporation, Dipesa SA, Spain) was used to get a spectrophotometric analysis. Normal reference range was considered to be between 0.18 and 1.20 mg / dl (13).

After this, a descriptive and comparative statistical analysis was performed with the obtained results. The two-tailed Student´s t test for independent samples with a confidence level of 99% (α, 0.01) and 95% (α, 0.05) was employed in the analysis of quan-titative variables.

## Results

All the patients in the study group had suffered at least 10 minor type aphthous lesions during the last year before the study. Thirteen patients (65%) suffered lesions almost continuously and without remissions. In 5 patients (25%), the periods free of lesions lasted from 1 to 3 months and only in 2 patients (10%) the periods lasted for more than 3 months. The most common locations for the lesions were the tongue (75%), buccal mucosa (70%) and labial mucosa (60%).

In relation to the triggers, 8 patients (40%) related having a significant increase in the number of ulcers during the periods in which they recognized having suffered situations of increased stress or anxiety.

Only 3 patients of RAS group (15%) presented increased levels of salivary cortisol.

**Figure 1:**
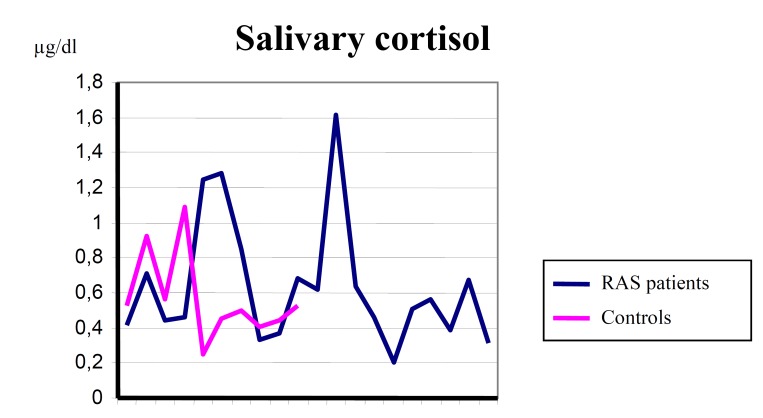
Salivary cortisol levels in patients with recurrent aphthous stomatitis (RAS) and controls.

None of the patients in the control group presented increased levels of salivary cortisol. The mean cortisol level was 0.64 mg / dl (mean deviation ± 0.36, range 0.2 to 1.62) for patients with RAS and 0.57 mg / dl (mean deviation ± 0 , 25, range from 0.25 to 1.09) for controls ( Table 1). After the statistical analysis, salivary cortisol levels were not statistically higher in patients with RAS (Fig. 1).

**Table 1:**
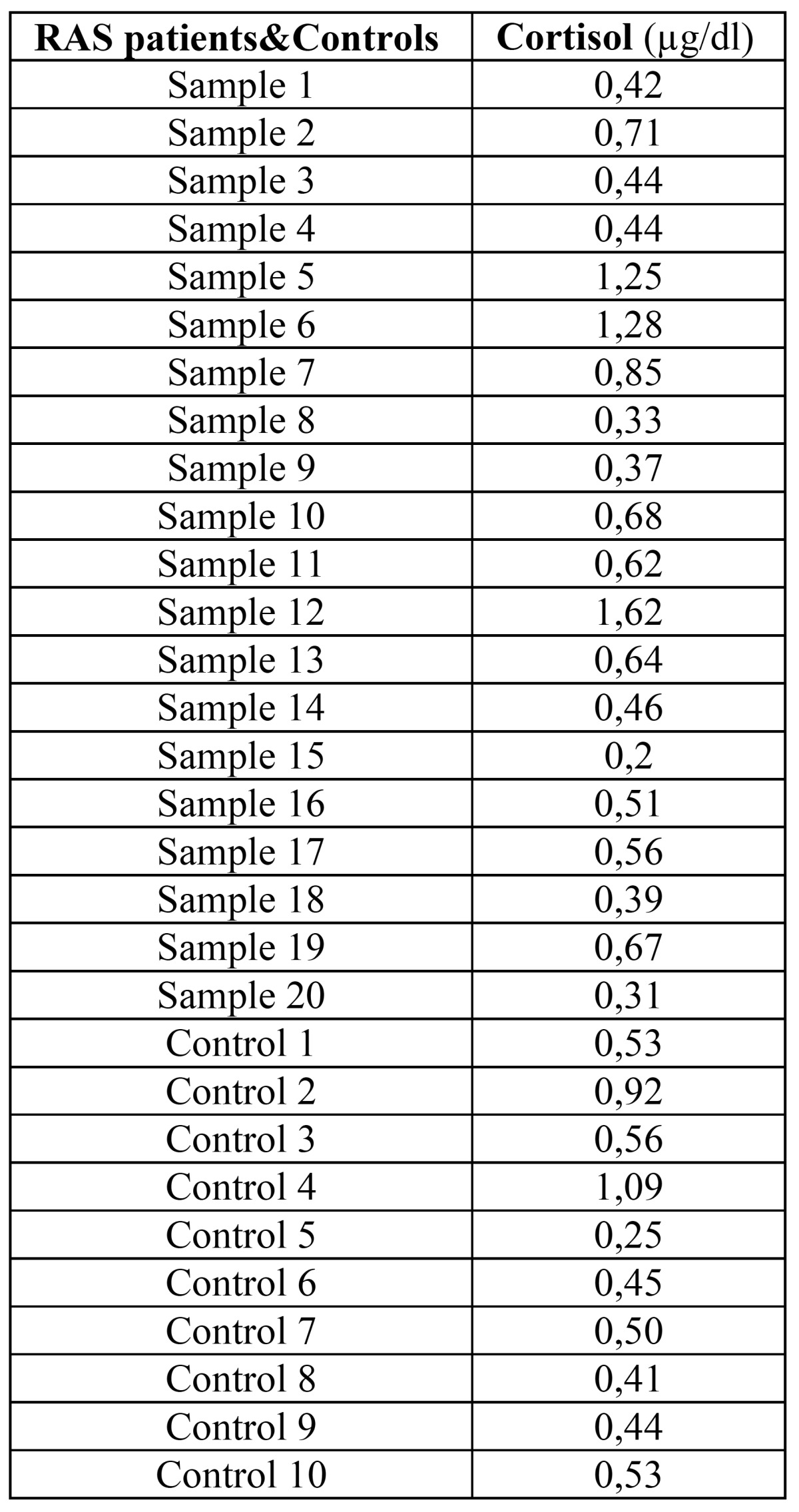
Results of the measurement of salivary cortisol levels in patients with recurrent aphthous stomatitis (RAS) and control patients.

## Discussion

It is well-known that some oral diseases may have a psychosomatic component (14). In RAS stress or anxiety, could act as precipitating factor causing the development of new lesions (1,2,9). The real role of this factor is still unknown but it can be probably related with the modifications that anxiety and stress produce in the oral mucosa, the increase of the parafunctional habits or the increase in some habits that causes microtraumatic injuries (1,2,9).

As a result, Soto-Araya and cols. (15) studied the prevalence and degree of stress, anxiety and depression among patients with different oral diseases such as burning mouth syndrome, oral lichen planus and RAS. Using the Recent experience test and the HAD scale (Hospital Anxiety scale and depression), they found increased levels of stress (61%) and anxiety (83%) among the 18 patients with RAS of their study group.

Later, Gallo and cols. (16) studied the stress levels through the questionnaire SSLVAS (Symptoms of Stress List, Institute of Psychology at the University of Sao Paulo, Brasil) in 55 patients with RAS and 25 healthy patients. Soto-Araya and cols. observed a significantly higher level of stress among patients with RAS too. However, they observed no statistically significant relationship between the clinical severity of RAS lesions and stress level.

Salivary cortisol has been shown in several studies (17,18) as a safe analytic tool, useful for detecting high stress or anxiety states. The production of cortisol by the adrenal glands is controlled by the secretion of the adrenocorticotropic hormone (ACTH) from the pituitary gland. ACTH secretion is regulated by the action of corticotropin releasing hormone (CRH), a hypothalamic hormone, which is stimulated by stress situations (15-18). Under normal conditions the secretion of ACTH is rhythmically produced synchronized with the sleep-wake cycle, so the secretion is maximal in the morning and minimal at midnight.This variation is known as circadian rhythm or cycle, and it allows us to maintain a high activity level during the day in contrast to the night.

Anxiety and depression states not only modify the physiological production of cortisol and its concentration in plasma, it also produces a proportional change in the concentration of cortisol in saliva (16,17).

In 1996 McCartan and cols. (9), compared the levels of salivary cortisol by radioimmunoassay, and the degree of anxiety by the HAD psychometric scale, between two different groups of patients with RAS. In the first group they included 12 patients with persistent oral lesions, no hematological alterations and no improvement in symptoms after oral empirical administration of vitamin B1 and B6. In a second group they included other 12 patients who had improved their oral symptoms after correcting hematological deficiencies, such as deficits of iron or vitamin B12. In this second group they also included patients who had had a positive response after empirical administration of vitamin B1 and B6. After a statistical analysis, they found a significantly higher level of anxiety and salivary cortisol among the patients in the first study group.

Later, Albanidou-Farmaki and cols. (19) also studied the possible association between anxiety, serum and salivary cortisol, and RAS. They selected a group of 38 patients with RAS and a control group of 38 healthy patients and carried out a determination of cortisol using chemiluminescent immunoassay. To determine the degree of anxiety they used a test consisting of two independent scales, one for the “Status Anxiety” (STAI-S) and other one for the “Trait Anxiety” (STAI-T).After analyzing the results they observed significantly higher cortisol levels, both in saliva and serum in the group of patients with RAS. Furthermore, levels of “trait anxiety” and “status anxiety” were also significantly higher in RAS patients than in healthy individuals.

The determination of salivary cortisol is a widely contrasted method to measure and quantify situations or states of stress and anxiety (6,10,11). In opposition to these previous works that measured salivary cortisol in patients with RAS, our results in this pilot study have not shown a significant increase in salivary cortisol. We don´t Know exactly what causes this difference, but we think they might be due to several reasons. The first one and the most obvious, is the limited sample size of the studies. Moreover, the populations studied are very different, have different epidemiological characteristics and might show differences in the prevalence of stress. In relation to the methodology employed, we do not know exactly how much could some of the important differences that we found have influenced in the results.

In our study, the saliva sample collection was performed in all cases during the active phase of the disease and was always carried out in the morning between 9 and 12 AM. We always collected unstimulated saliva. In the study of Albanidou-Farmaki and cols. (18), we don´t know if the patients had active lesions at the time of sample collection. In their study the samples were collected from stimulated saliva (with 10% citric acid) and inclusion and exclusion criteria of patients were different. Processing and technical methods and materials used by them were different too.

In the study of McCartan and cols. (9), sample collection was performed between 1 and 2 PM and using saliva stimulated with a 10% citric acid solution. While in their first group of the study samples were collected during the active phase of the disease in the second it is unclear. We can conclude reading their article that they collected the samples in this second group in patients free of lesions at that moment. In this study, inclusion criteria and processing methods and materials used were different from ours too.

Although our overall results do not show a significant increase of salivary cortisol, we found that patients with the higher salivary cortisol concentrations suffered more severe clinical forms of the disease, according to the number, size and recurrence of lesions.

As a conclusion of this pilot study we can remark that we could not observe the existence of significantly higher salivary cortisol levels among the patients with RAS in our region during periods of active disease than in healthy individuals.

Due to the differences observed between our results and those obtained by other authors, we believe that further studies are needed in other populations with larger sample sizes. In this way we will be able to determine the real role of salivary cortisol and the real importance of stress and anxiety as triggers in the pathogenesis of the RAS.

## References

[1] Ship JA (1996). Recurrent aphthous stomatitis. An update. Oral Surg Oral Med Oral Pathol Oral Radiol Endod.

[2] Scully C (2006). Clinical practice. Aphthous ulceration. N Engl J Med.

[3] Miller GE, Chen E, Zhou ES (2007). If it goes up, must it come down? Chronic stress and the hypothalamic-pituitary-adrenocortical axis in humans. Psychol Bull.

[4] Maguire AM, Ambler GR, Moore B, McLean M, Falleti MG, Cowell CT (2007). Prolonged hypocortisolemia in hydrocortisone replacement regimens in adrenocorticotrophic hormone deficiency. Pediatrics.

[5] Ahn RS, Lee YJ, Choi JY, Kwon HB, Chun SI (2007). Salivary Cortisol and DHEA Levels in the Korean Population: Age-Related Differences, Diurnal Rhythm, and Correlations with Serum Levels. Yonsei Med J.

[6] Gozansky WS, Lynn JS, Laudenslager ML, Kohrt WM (2005). Salivary cortisol determined by enzyme immunoassay is preferable to serum total cortisol for assessment of dynamic hypothalamic-pituitary-adrenal axis activity. Clin Endocrinol (Oxf).

[7] Hashem AA, Claffey NM, O'Connell B (2006). Pain and anxiety following the placement of dental implants. Int J Oral Maxillofac Implants.

[8] Weekes N, Lewis R, Patel F, Garrison-Jakel J, Berger DE, Lupien SJ (2006). Examination stress as an ecological inducer of cortisol and psychological responses to stress in undergraduate students. Stress.

[9] McCartan BE, Lamey PJ, Wallace AM (1996). Salivary cortisol and anxiety in recurrent aphtous stomatitis. J Oral Pathol Med.

[10] Gozansky WS, Lynn JS, Laudenslager ML, Kohrt WM (2005). Salivary cortisol determined by enzyme immunoassay is preferable to serum total cortisol for assessment of dynamic hypothalamic-pituitary-adrenal axis activity. Clin Endocrinol (Oxf).

[11] Rai B, Kaur J, Anand SC, Jacobs R (2011). Salivary stress markers, stress, and periodontitis: a pilot study. J Periodontol.

[12] Rivera-Gómez B, Hernández-Vallejo G, Arriba-de la Fuente L, López-Cantor M, Díaz M, López-Pintor RM (2006). The relationship between the levels of salivary cortisol and the presence of xerostomia in menopausal women. A preliminary study. Med Oral Patol Oral Cir Bucal.

[13] Roberts RF, Roberts WL (2004). Performance characteristics of five automated serum cortisol immunoassays. Clin Biochem.

[14] Chaudhary S (2004). Psychosocial stressors in oral lichen planus. Aust Dent J.

[15] Soto-Araya M, Rojas-Alcayaga G, Esguep S (2004). Association between psychological disorders and the presence of Oral lichen planus, Burning mouth syndrome and Recurrent aphthous stomatitis. Med Oral.

[16] Gallo Cde B, Mimura MA, Sugaya NN (2009). Psychological stress and recurrent aphthous stomatitis. Clinics (Sao Paulo).

[17] Eller NH, Netterstrom B, Hansen AM (2006). Psychosocial factors at home and at work and levels of salivary cortisol. Biol Psychol.

[18] Smyth J, Ockenfels MC, Porter L, Kirschbaum C, Hellhammer DH, Stone AA (1998). Stressors and mood measured on a momentary basis are associated with salivary cortisol secretion. Psychoneuroendocrinology.

[19] Albanidou-Farmaki E, Poulopoulos AK, Epivatianos A, Farmakis K, Karamouzis M, Antoniades D (2008). Increased anxiety level and high salivary and serum cortisol concentrations in patients with recurrent aphthous stomatitis. Tohoku J Exp Med.

